# Growth, maturity, reproduction, and life expectancy in *ex-situ* pacific walruses (*Odobenus rosmarus divergens*)

**DOI:** 10.1186/s40850-022-00158-1

**Published:** 2022-12-05

**Authors:** Todd Robeck, Etsuko Katsumata, Kazutoshi Arai, Gisele Montano, Todd Schmitt, Stacy DiRocco, Karen J. Steinman

**Affiliations:** 1 Zoological Operations, SeaWorld Parks and Entertainment, 7007 SeaWorld Drive, Orlando, FL USA; 2 Kamogawa Sea World, 1464-18 Higashi-Cho, Kamogawa, Chiba Japan; 3 Species Preservation Laboratory, SeaWorld San Diego, 2595 Ingraham Rd, San Diego, CA USA; 4 SeaWorld San Diego, 500 SeaWorld Drive, San Diego, CA USA; 5 SeaWorld Orlando, 7007 SeaWorld Drive, Orlando, FL USA

**Keywords:** Walrus, Reproduction, False pregnancy, Embryonic diapause, Gestation, Survivorship

## Abstract

**Background:**

Pacific walruses are found in Arctic regions of the Chukchi and Bering Sea where rapid changes in environmental conditions resulting in loss of sea ice are occurring. Therefore, accurate life history data are crucial for species management plans and longitudinal data collected over the lives of individual walruses housed in zoos and aquaria provide otherwise difficult to obtain biological information.

**Results:**

While similar at birth, Gompertz regression curves indicated that males grew faster than females (*p* < 0.0001) in weight (99 kg vs 57.6 kg/y) and length (26.9 cm vs 26.3 cm/y) with physical differences being detected by age 3 for weight and age 7 for length. Males reached adult weight at 13.5 ± 3.3 y and females by age 12.3 ± 2.3 y. The mean age at first ovulation and at first conception occurred at 8.8 y and 9.6 y. Greater than 75% of all conceptions and calving occurred between February and March and from May to June, respectively. Mean gestation lasted 423 d and false pregnancies lasted at least 169 d with a decrease (*p* < 0.05) in serum progesterone concentration between false pregnancy and pregnancy occurring within 6 months after ovulation. Based on these results, we estimated embryonic diapause to last from 120 to 139 days, and fetal growth last ~ 284 days. All males older than 8 y had an increase in serum testosterone and body weight that was highest in February and lowest in July. Overall, no differences were observed between male and female survival, with a mean (± SEM) life expectancy of 19.5 ± 1.5 y, respectively. Currently, the oldest male and female captive walruses are 40 and 43 y, respectively.

**Conclusions:**

Data provided herein include details of life history characteristics of zoo and aquaria housed walruses that are useful for wild population recovery models. In particular, results on survivorship and the identification of the most vulnerable period for calf survival can help with model development and suggests that for recovery to occur birthing locations for this species must be protected.

**Supplementary Information:**

The online version contains supplementary material available at 10.1186/s40850-022-00158-1.

## Background

The Pacific walrus (*Odobenus rosmarus divergens*) inhabits the Arctic and subarctic portions of the Pacific Ocean mostly along the edge of the sea ice in the Bering Sea during the winter and onshore during the summer in the Bering and Chukchi Seas [1–3]. Breeding occurs primarily in January and February with calving and nursing occurring on sea ice from April to June [3]. Pacific walrus distribution is limited by the location of their benthic food sources, primarily bivalves, which are only found in relatively shallow water (< 100 m) [4, 5]. In addition to relatively shallow water, walruses require haul-out substrates, whereby, they can get out of the water for resting after foraging, for socialization, calving and nursing [2, 3]. In Pacific walruses, evidence suggest they are normally associated with sea ice all year long [2, 4]. In winter they are typically found offshore in areas of unconsolidated ice in the Bering Sea, with open leads for breathing and feeding, and during the summer they follow sea ice as it recedes north into the Chukchi sea [1, 4, 6]. Recently ice free months in the Chukchi sea during the summer have caused walruses to haul out on shore [2]. During periods when no sea ice is available, it appears walruses have reduced food intake due to spending more time traveling back and forth between benthic food sources and on-shore haul-out locations [6]. The lack of sea ice can be particularly dangerous for females and young calves who due to lack of sea ice are forced to haul out on land [2]. For example, little to no availability of sea ice can result in higher densities of on shore animals, which have been linked to an increase in intraspecific trampling of juveniles, possibly greater predation and increased rates of disease and parasitism [2, 7–10]. Therefore, rapid environmental changes could not only disrupt walrus food sources but challenge their ability to behaviorally and physiologically adjust [11, 12].

In order to determine the population health of any wild species and its ability to adjust to changing conditions knowledge of several life history parameters, including individual animal age estimates, life expectancy and reproductive history are needed. Obtaining these data from wild populations is challenging due to limited access to animals and the inability to observe animals throughout their lifespan. As a result, much of the available data for walruses, and in particular reproductive history data, have come from photo surveys and post mortem examinations of harvested animals [3, 13]. Growth rate curves and their relationship to age have been demonstrated in both wild [3, 14] and captive animals [3, 15]; however, these data from captive animals are based on 8 and 2 animals, respectively. Based on analyses of reproductive organs from harvested animals, wild male Pacific walruses are estimated to reach sexual maturity by age 10 [3]. In a captive male Pacific walrus, his first successful copulation was observed at 9 y [15]. The majority (~ 68%) of wild, female Pacific walruses are estimated to reach sexual maturity at approximately ~ 6 y with almost all (~ 98%) mature by 8 y [3, 16, 17]. The first copulatory behavior in two captive females occurred at 6 and 9 y, respectively [15]. The walrus breeding season occurs from January through March [3], with a gestation length of about 15 months, including a period of delayed implantation or embryonic diapause [3, 15]. The inter-birth interval has been estimated from 2 to 3 y [3, 18, 19]. The maximum age of successful reproduction in females is approximately 30 y [13].

Natural mortality rates and life span data for wild Pacific walruses are difficult to determine because the opportunity to observe deaths or examine causes of death are rare [3]. Maximum longevity is estimated to be about 40 y [3], with anecdotal reports of a few females reaching 36 y of age [20]. Thus, the species is typically considered to be long-lived and previously, adult natural mortality rates, excluding calf mortality, have been estimated to be low (about 1 to 2% [20]), so that at the population level, mortality was most influenced by the subsistence harvests [13]. While harvested animals provide cross sectional data on age structure, hunter bias can skew the estimates toward older animals affecting demographic estimates [17, 20]. As a result, accurate calf and juvenile survival data are also important additions for the development of precise population models. However, collection of age specific survival rates is difficult due to the widespread distribution of walruses that are inaccessible during a large part of the year. In contrast, walruses housed in zoos and aquaria are not subjected to many of these variables and can provide better data for the development of predictive models of survival.

Zoos and Aquaria are typically free from environmental, ecological, and anthropogenic pressures and may serve as a control for comparison to wild populations. Furthermore, the ability to acquire detailed known life history data, such as growth, reproduction, and longevity data from such a control sample can be easily obtained. Therefore, our objective was to evaluate the life history of captive Pacific walruses from four locations over a period of 40 y. The specific aims were to: 1) evaluate reproductive history, including age at sexual maturity and gestation length; 2) compare and contrast progesterone concentrations during pregnancy and false pregnancy in an attempt to define the length of diapause and embryonic growth; 3) describe annual changes in male reproductive activity using serum testosterone and seasonal changes in body weight; 4) establish growth models for both sexes, including body length and weight; and 5) determine annual survival rates and life expectancy for this species.

## Results

### Growth and physical maturation

**Fig. 1:**
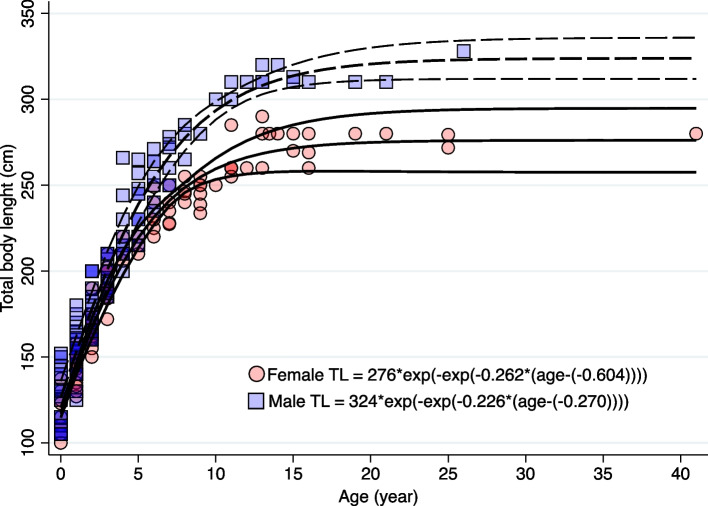
Exponential relationship, as described by Gompertz regression, in male (*n* = 11) and female (*n* = 15) walruses between age (y) and total length (cm). Males grew significantly (**χ**^**2**^** = **29.7 *p* < 0.0001) faster than females, with an absolute growth rate (R_G_) of 26.9 cm/year for males compared to 26.6 cm/year for females. Males reached asymptote length by age 13 (95% CI: 309.7 to 338.0 cm), and females (95% CI: 255.9 to 296.4 cm) by age 9.8 y

Details for walruses used in the study are listed in Tables 1 & 2. For age at length, no difference was detected in mean (± SEM) length of yearlings between males (125.5 ± 3.3 cm, 95% Confidence interval (CI): 118.6 to 132.2 cm) and females (116.0 ± 4.9 cm, 95% CI: 105.9 to 126.1 cm). Across all ages, however, males grew in length faster (TL, **χ**^**2**^** = **29.7, *p* < 0.0001) than females of similar ages (Fig. 1). Because growth rates differed between males and females, we fit separate Gompertz growth curves for each sex. Gompertz growth curves identified a strong relationship between length at age for males (Adj *R*^2^ = 0.996, mean squared error (MSE) = 13.9 cm) and females (Adj *R*^2^ = 0.998, MSE = 9.79 cm) (Table 3). The absolute growth rate for males was 26.9 cm/year and for females was 26.6 cm/year (Table 3). Only one male had total length data recorded within a year before and after he reached the lower end of the 95% CI (309.7 to 338.0 cm) for the predicted adult lengths (upper asymptote, 323 cm). This male reached 310 cm by age 11 y and he attained his final adult length of 320 cm by 13 y. Four females reached the lower end of the 95% CI (255.9 to 296.4 cm) for adult length (upper asymptote, 276.1 cm) by a mean age of 9.8 ± 2.3 y (Table 3). Males were longer (cm, *p* = 0.013) than females by age 8 y (Male: 95% CI 272 to 301 cm; Female: 95% CI 238 to 264 cm, Fig. 1).

**Table 1:** Details of female walruses and their contribution to data within this research

ID	Facility	DOB (m/d/y)	Wild (W) Captive (CB)	Growth data (age range [y], n)	Progesterone (age range [y], n)	Ovulations^a^	Pregnancies
1	KSW	5/01/1983	W	0 to 21, 186	16 to 17, 6	5	4
2	KSW	5/03/2000	CB	0 to 16, 317	7 to 16, 21	4	4
3	KSW	5/20/2007	CB	0 to 9, 133	5 to 11, 14	2	2
4	KSW	5/05/2010	CB	0 to 9, 274	6 to 8, 6	1	0
5	KSW	5/01/2016	CB	0 to 3, 111			
6	SEAS	5/01/1968	W		17 to 21, 13	2	1
7	SEAS	5/17/1974	W	0 to 2, 75			
8	SEAS	5/01/1978	W	0 to 1, 33			
9	SEAS	5/01/1978	W	0 to 38, 559	12 to 36, 36	3	1
10	SEAS	5/01/1978	W	0 to 41, 899	8 to 40, 67	5	0
11	SEAS	5/01/1978	W	0 to 2, 68			
12	SEAS	4/21/1981	W		12, 6	1	1
13	SEAS	5/15/1981	W		8 to 29, 43	9	4
14	SEAS	6/01/1983	W	24 to 25, 38		2	2
15	SEAS	6/22/1987	CB	10 to 28, 856	4 to 29, 26	7	5
16	SEAS	5/01/1994	CB				
17	SEAS	5/01/2003	CB	0 to 16, 1223	8 to 16,21	3	2
18	SEAS	6/03/2017	CB	0 to 2, 248			
19	SEAS	7/03/2019	CB	0 to 0.2, 22			

*Facility KSW* Kamogawa Sea World, *SEAS* SeaWorld Parks, *DOB* date of birth

^a^Ovulations = represents total number of pregnancies and false pregnancies where blood samples for progesterone were collected with sufficient frequency to define the event. Note this does not represent all of the potential ovulations and false pregnancies that may have occurred within each animals’ reproductive history because in some years no serum was available for retrospective hormone analysis and/or sample were not collected frequently enough throughout the year to detect an ovulation

**Table 2:** Details of male walruses and their contribution to data within this research

ID	Facility	DOB (m/d/y)	Wild (W) Captive (CB)	Growth data (age range [y], n)	Testosterone (age range [y], n)	Sire (Y/N)
20	KSW	5/1/1983	W	1 to 25, 237	24.3 to 33.2, 44	Y
21	KSW	5/27/1997	CB	0 to 6, 66		Y
22	KSW	6/1/2003	CB	0 to 9, 180		N
23	KSW	4/23/2013	CB	0 to 4, 81		N
24	KSW	4/19/2018	CB	0 to 2, 114		N
25	SEAS	5/1/1978	W	0 to 2, 11 to 29, 311		Y
26	SEAS	5/6/1978	W	0 to 1, 18 to 31, 167		Y
27	SEAS	1/15/1982	W		27.0 to 37.4, 38	Y
28	SEAS	6/21/1993	CB	0 to 26, 941	24.1 to 26.7, 24	Y
29	SEAS	5/1/2012	W	3 to 8, 296	4.2 to 7.4, 21	N
30	SEAS	5/1/2017	W	0 to 2, 127		N

*Facility KSW* Kamogawa Sea World, *SEAS* SeaWorld Parks, *DOB* date of birth, *Sire, Y* Proven sire

**Table 3:** Parameter estimates, standard errors and 95% confidence intervals (CI) for allometry of weight (kg) and total length (TL, cm) 3-parameter Gompertz growth equation as follows: Weight or TL = b1*exp(-exp(-k_G_*(age-b3))). Standard errors and CI were generated using Bootstrap (BS) estimates for weight or Jacknife (JN) estimates for total length

Parameter	Parameter Estimates	BS or JN Std Errors^a^	95% Confidence Intervals
Age (y) by kg (all females)	Adj *R*^2^ = 0.989 MSE = 73.9 kg		
A	946.3	65.97	816.9 to 1075.6
k_G_	0.1656	0.0298	0.1071 to 0.2242
T_i_	4.21 (y)	0.55	3.09 to 5.32
Age (y) by kg (all males)	Adj *R*^2^ = 0.988, MSE = 106 kg		
A	1381	93.5	1197.8 to 1564.4
k_G_	0.1948	0.0254	0.1449 to 0.2447
T_i_	4.34 (y)	0.45	3.47 to 5.23
Age (y) by TL (all females)	Adj *R*^2^ = 0.998		
A	276.1 (cm)	10.2	255.9 to 296.4
k_G_	0.2622	0.0344	0.1843 to 0.3401
T_i_	-0.6037	0.085	-0.797 to -0.410
Age (y) by TL (all males)	Adj *R*^2^ = 0.996		
A	323.9 (cm)	6.14	309.7 to 338.0
k_G_	0.2256	0.0209	0.1774 to 0.2739
T_i_	-0.2699	0.2228	-0.7838 to 0.24440

^a^Bootstrap replacement [1000 reps] was used within Gompertz weight by age predictions, variance–covariance matrix estimates were set for cluster option around animal id. Due to small sample size, Jacknife variance–covariance estimates (VCE) were used with TL data

^b^A = upper asymptotic Weight or TL can be used to approximate the adult weight of the population, k_G_ is the growth-rate coefficient, T_i_ is the timepoint at curve inflection (Figs. 1 and 2)

**Fig. 2:**
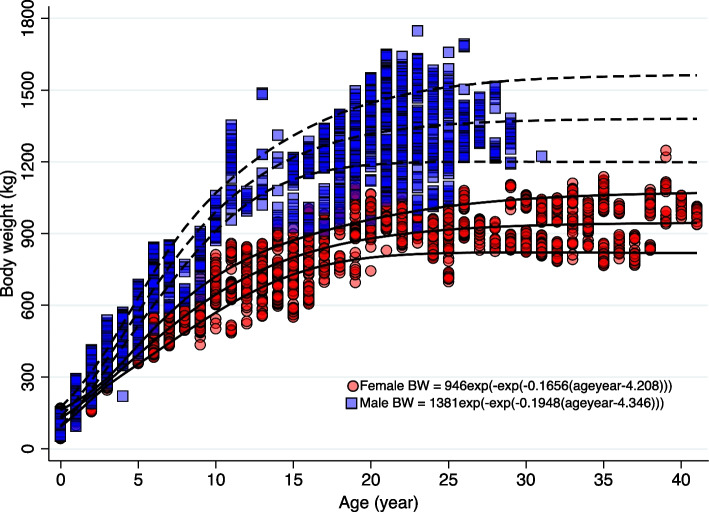
Exponential relationship, as described by Gompertz regression, in male (*n* = 11) and female (*n* = 15) walruses between age (y) and weight (kg). Males gained weight at a significantly (**χ**^**2**^** = **34.9, *p* < 0.0001) faster rate than females, with an absolute growth rate (R_G_) of 99 kg/year for males compared to 57.6 kg/year for females. Males reached asymptote weight by age 13.5 (95% CI: 1198 to 1564 kg), and females (95% CI: 816 to 1075 kg) by age 12.3 y

No differences (*p* = 0.128) were detected in mean (± SEM) weight between males (65.5 ± 5.1 kg, 95% CI: 54.6 to 76,0.4, n = 4) and females 60.5 ± 3.6 kg (95% CI: 49.3 to 62.5, n = 11) walruses up to age 3 y. Across all ages, males grew significantly heavier with age (male WT, **χ**^**2**^** = **34.9, *p* < 0.0001) than females (Fig. 2). Separate Gompertz curves identified a strong relationship between weight with age for both males (Adj *R*^2^ = 0.989, MSE = 105 kg) and females (Adj *R*^2^ = 0.989, MSE = 73 kg cm, Table 3). Males had an absolute growth rate of 99 kg/y and reached the lower end of the 95%CI (1198 to 1564 kg) for adult weight (upper asymptote, 1381 kg) at a mean age of 13.5 ± 3.3 y (Table 3). Females had an absolute growth rate of 57.6 kg/y and reached the lower end 95% CI (816 to 1075 kg) for adult weight (upper asymptote, 946 kg) at a mean age of 12.3 ± 2.3 y (Table 1). Males were larger (kg, *p* = 0.021) than females by age 2 (male: 95% CI 275 to 313 kg; female: 95% CI 228 to 262 kg). Predicted growth in weight for male and female juveniles ages 1 to 4 y is provided in Supplementary File 1.

### Female reproductive characteristics

**Fig. 3:**
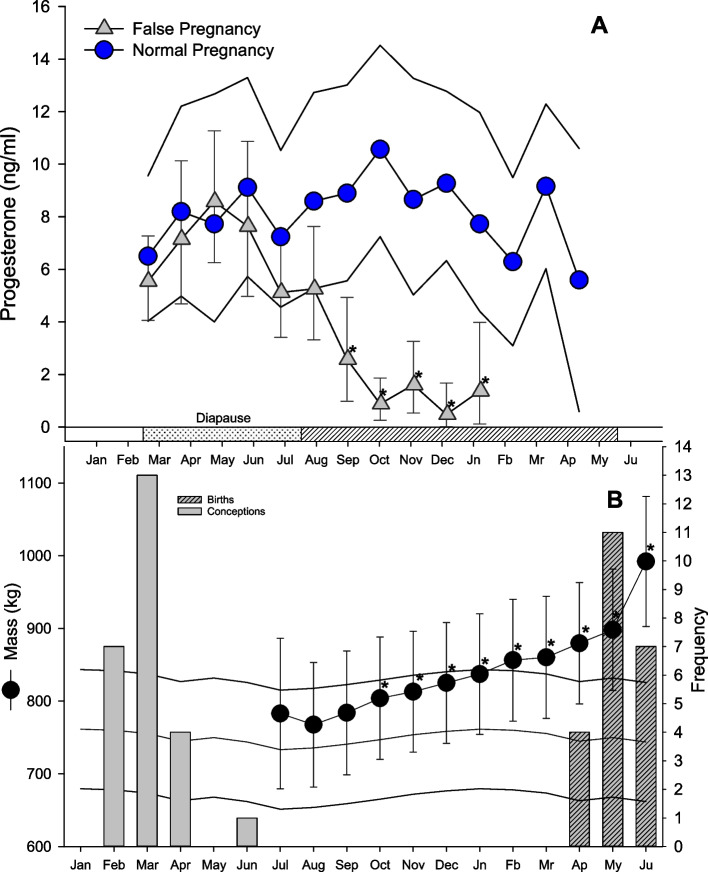
Female walrus reproductive patterns. **A** The mean (± 95% CI, solid black line) monthly serum progesterone (ng/ml) concentrations during pregnancy and false pregnancy. Diapause is proposed to occur from March to July. Initiation of embryonic growth is estimated to occur in August whereby mean progesterone concentrations drop significantly for false pregnant females. Significant differences in concentrations between pregnant and false pregnant females are noted (*). **B** Patterns of conceptions, births, and weight (Kg). The 95% CI for the mean monthly annual changes in weight are demonstrated by two parallel lines. Mean monthly weight in pregnant females that is significantly different from non-pregnant females is marked (*)

During this study period a total of 26 pregnancies were recorded (Table 4). First ovulation occurred at a mean age of 8.6 y (range 4.8 to 12.7 y), with first conception at 9.6 y when females were 95% of their adult weight. The oldest female to give birth was 28 y, and the oldest to ovulate was 36 y. No evidence of more than one ovulation per season was observed during the sampling period and all ovulations were either fertile or resulted in a prolonged luteal phase (i.e., false pregnancy). Gestation length was 423 d with an overall 2.6 y calving interval (Table 4). The calving interval, for females with consecutive calves that were parent-raised, was always 3 y (*n* = 6). The calving interval, for females that calved after a stillbirth or after a calf that they did not nurse, averaged 2.3 ± 0.5 y (*n* = 9). One female which did not have access to a breeding male for a number of years and was not used in the analysis. Conception dates that could be estimated (23 of 26) occurred from February to June, although none occurred in May, with most (80%) occurring in February and March (Fig. 3B). Full term births occurred from April through June, with the majority (77%) occurring in May and June (Fig. 3B). No differences (T_df=22_ = 0.89, *p* = 0.38) were observed between birth dates in relation to the spring equinox at SEAS facilities (60.1 ± 7.9 d) and those at KSW (51.6 ± 5.4 d). Non-term births included 4 sets of twins (15.4%) and one non-twin abortion (3.8%). The same female was responsible for three sets of twins and the one non twin abortion; however, her first pregnancy produced a viable calf. Sampling frequency for progesterone did not permit accurate determination of the length of false pregnancies, however, the minimum length (the length from ovulation to last elevated progesterone sample) could be determined and averaged 230 d ± 34 d (median = 234, range 169 to 293, Fig. 3A). False pregnancy accounted for a third (32%) of the periods during which elevated progesterone was detected in adult female walruses (Table 4).

**Table 4:** Reproductive maturity and pregnancy outcome for captive female Pacific walruses from 1980 through 2019

**Reproductive parameter**	**Mean ± SD**	**Median (*range*)**	**Observations**
Age (y) at first ovulation	8.6 ± 2.4	7.8 (*4.8 to 12.7*)	12
Age (y) at first conception	9.6 ± 2.5	8.9 (*5.8 to 13.8*)	9
Weight (kg) at first conception	658 ± 111	649 (*488 to 845*)	6
Age (y) at maximum weight (kg)	10.2 ± 0.9	11 (*8 to 11*)	5
Percent of adult weight at first conception	0.95 ± 0.08	0.96 (*0.76 to 1.1*)	6
Age (y) of all conceptions	14.7 ± 4.3	14.4 (*5.9 to 28.5*)	26
Min false pregnancy length	231 ± 34	234 (*169 to 293*)	12
Gestation length (d)	423 ± 13.4	425 (*394 to 441*)	11
Calving Interval	2.6 ± 0.5	3 (*2 to 3*)	15
**Reproductive event outcomes**			
**Parameter**	**Total number (%)**		**% of Pregnancies**
False pregnancies	12 (31.6)		
Twins	4 (10.5)		15.4
Abortion	1 (0.03)		3.8
Stillbirth	4 (10.5)		15.4
Live births	17 (44.7)		65.4
Total Events	38 (100)

### Female weight fluctuations

Across all non-pregnant, non-lactating adult females (> 8 y) month had an effect (**χ**^**2**^** = **531, *p* < 0.0001) on body weight, with peak mean weight occurring in January at 761 kg (95% CI 679 to 843 kg). The mean weight in January was 28 kg higher by month than the lowest monthly weight in July of 733 kg (95% CI 651 to 815 kg). Weight for females fluctuates seasonally whether they are pregnant or not, therefore, we also compared monthly weight within months of gestation and determined that, for pregnant females, mean peak weight occurred in June, just prior to birth at 992 kg (95% CI 902 to 1081 kg). This pre-birth weight was 249 kg higher than (*p* < 0.05) mean June weight in non-pregnant animals. Further inter-month comparisons indicated that a significant difference in weight between pregnant and non-pregnant females was first observed in October and continued until parturition (Fig. 3B).

### Progesterone concentrations (P4) during pregnancy and false pregnancy

A total of 260 serum samples were analyzed for progesterone (P4) from adult non-pregnant (*n* = 68), false pregnant (*n* = 116) and pregnant females (*n* = 76). P4 was square root transformed for all statistical comparisons. Combined across all month’s post ovulation (MPO), mean P4 was greater (*p* < 0.001) in pregnant (9 ng/ml, 95% CI 8 to 9.9 ng/ml) verses false pregnant (FP, 5.3 ng/ml, 95% CI 4.4 to 6.1 ng/ml) females. In addition, both groups had increased (*p* < 0.001) P4 concentrations when compared against non-pregnant adult females (baseline, 0.45 ng/ml, 95% CI 0.38 to 0.52 ng/m).

During pregnancy, no mean month post conception (MPC) P4 concentrations were significantly different than any other MPC concentrations. False pregnant females had an early P4 peak that occurred in May (MPO 2) at 8.6 ng/ml (95% CI 6.3 to 11.3 ng/ml) with concentrations decreasing in a relatively linear fashion every month thereafter returning to less than 2 ng/mL by October (MPO 7). For direct intra-MPO comparisons between pregnant and false pregnant females, P4 began decreasing by July for FP females, and concentrations were significantly different (p < 0.05) compared with pregnant females by September (MPO 6). Based on these results, we subjectively defined the period of embryonic diapause from March 15 through July (~ 139 d), implantation and gestation from August 1 to May 11^th^ of the following year (~ 284 d). For comparisons and based on the previously published premise that the period of embryonic diapause is followed by an increase in P4 for pregnant females and a concomitant decrease in P4 for FP females [21, 22], we can then divide the post-ovulatory periods into pre- and post-implantation or activation. Therefore, we considered the initial peak observed between both groups (pregnant vs FP) as the luteal phase and the period of embryonic diapause and the second period as fetal growth for pregnant females and luteal regression for FP animals. By doing so, we can break up the time period to pre- (PrI) and post-implantation (PI). Within these groups, we found that mean PI progesterone for FP (2.3 ng/ml, 95% CI 1.4 to 3.4 ng/ml) was reduced (*p* < 0.05) compared against to all other groups (FP PrI: 6.6 ng/ml, 95% CI 5.2 to 8.1 ng/ml; PG PrI: 7.5 ng/ml, 95% CI 5.7 to 9.6; PG PI: 8.4 ng/ml, 95% CI 6.8 to 10.3 ng/ml). No differences amongst the other three groups were observed (*p* < 0.05, Fig. 3A).

### Male reproductive characteristics and serum testosterone (T) characteristics

**Fig. 4:**
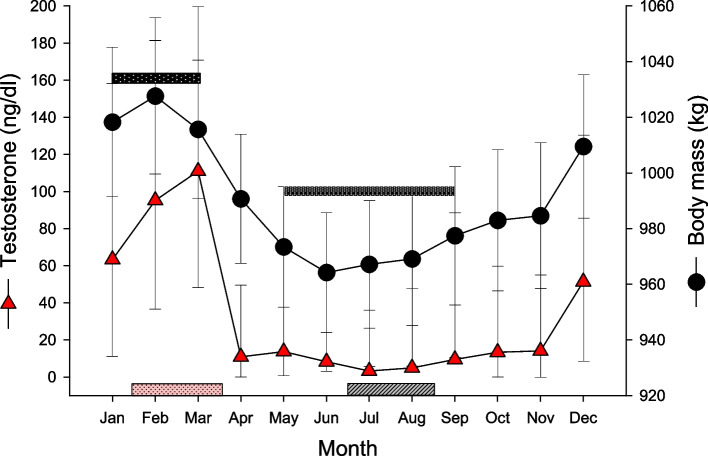
Mean (± 95% Confidence interval) monthly serum testosterone (red triangles) and body weight (black circles) in the male Pacific walrus (*n* = 11). Months that were significantly (p < 0.05) different within each analysis are highlight with bars. The black horizontal bars on top of the graph represent months during which weights were significantly different from each other, while the pink and grey bars along the x -axis represent months whereby testosterone concentrations are significantly different

Sample sizes across all age groups and months were too few to definitively determine age at reproductive maturation, however, for one male, serum T was first detected above assay baseline (0.1 ng/dl) at age 6 y, with peak concentrations of 45.34 ng/dl being detected during February at age 6.7 y. The earliest age at which a male sired a calf was age 9 y. The mean age for known sires was 12.2 ± 2.5 y (median: 11.9 y, *n* = 8). For males > age 7 y, testosterone fluctuated seasonally (χ^2^ = 52, *p* < 0.0001) with significant peaks (*p* < 0.05) occurring in February (95 ng/dl, 95% CI: 37 to 181 ng/dl) and March (110 ng/dl, 95% CI: 48 to 199 ng/dl) when compared to nadir during July (3.2 ng/dl, 95% CI: 0.1 to 36 ng/dl) and August (4.9 ng/dl, 95% CI: 0.1 to 47 ng/dl, Fig. 4).

### Male seasonal weight fluctuations

Across all adult males (> 8 y), significant changes (**χ**^**2**^** = **240, *p* < 0.0001) in monthly weight were observed with a mean peak weight in January of 1018 kg (95% CI 991 to 1045 kg). Both January and February were increased (*p* < 0.05) compared to July, with the mean weight in January being 285 kg heavier than the mean weight in July (733 kg, 95% CI 651 to 815 kg, Fig. 4).

### Survival and longevity

**Fig. 5:**
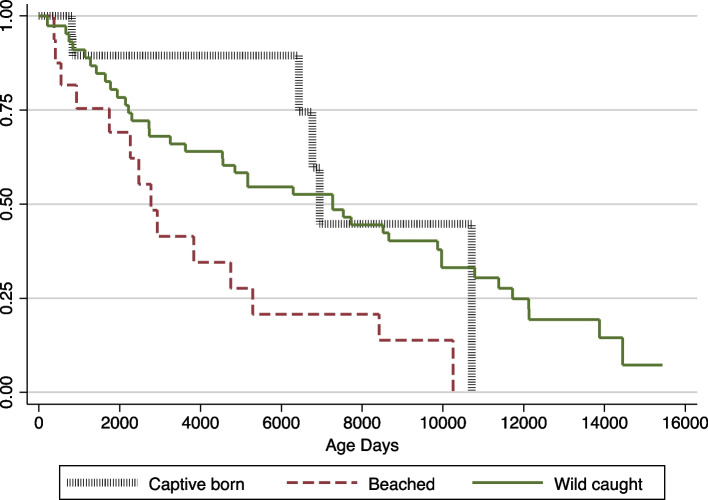
Kaplan–Meier survival curves for the proportion of Pacific walrus calves alive over time (d) for captive born (*n* = 39), beached (calves rescued outside of the breeding season, *n* = 20) and wild caught (calves collected during the calving season as neonates, *n* = 59)

Survival rates for all zoo and aquaria born term calves (live and stillborn) from birth to 90 d (subgroup 1) and birth to 365 d (subgroup 2) were 52.6% and 52.3%, respectively. Survival rates for term calves born alive only at 90 d (subgroup 3) and 365 d (subgroup 4) were 86.2% and 86.1%, respectively. Finally, survival rate for older calves from 90 to 365 d (subgroup 5) was 100%. Overall survivorship for all walruses, wild or captive born, after ~ 6 months of age had a median and mean (± SEM) life expectancy of 18.5 y and 19.5 ± 1.5 y, respectively. The current ages of the oldest male and female captive walrus, both of whom are currently living, are 40 and 43 y, respectively. The median and mean (95% CI) life expectancy of males and female walruses collected as neonates (wild caught), orphaned (beached) and brought into a facility for care, or captive born were as follows: beached (*n* = 20), 7.6 and 10.5 y (6.0 to 15.0 y); captive born (*n* = 39), 19.3 and 21.9 y (17.0 to 26.9 y); and wild caught (*n* = 59), 19.9 and 20.6 y (16.7 to 24.6 y). Comparisons found differences (χ^2^ = 11.1, *p* = 0.004) between survivorship curves from each group (Fig. 5). Further pairwise comparisons indicated that beached animals had significantly reduced survival compared against both wild caught (χ^2^ = 7.3, *p* = 0.007) and captive born (χ^2^ = 9.0, *p* = 0.003) animals, with no differences (χ^2^ = 1.93, *p* = 0.16) being detected between captive born and wild caught animals. No differences (*p* > 0.05) were detected between male (median = 14.2 y, mean = 17.4 y, 95% CI: 13.0 to 21.8 y) and female (median = 19.9 y, mean = 20.7 y, 95% CI: 16.5 to 25.0 y) survival across all groups and within any subgroup tested. The mean annual survival rate as determined from life tables (Supplementary File 2) from 6 y through 29 y was 96.2% (range 0.83 to 1.0).

## Discussion

Using data collected over 40 y from 30 Pacific walruses in human care from four zoological institutions, we described life history parameters for this species including growth, reproductive maturity, timing of reproduction, and life expectancy.

### Maturity

Based on spermatogenesis, in the wild male Pacific and Atlantic walrus the onset of puberty commences between 5 to 8 y with most males reaching sexual maturity between 10 and 13 y [3, 15, 23–25]. Similarly, a report for a captive walrus has found that semen was first produced at age 5 y, however, the male did not successful breed until age 10 y [15]. Using serum testosterone, we observed significant increases in hormone production by age 6 y, with apparent mature concentrations of testosterone (> 50 ng/dl) occurring as early as 8 y. Within our group of males, the mean age at first successful copulation was 12.2 y, similar to a report for wild walruses that used testicular growth and determined that sexual maturity in males occurred around 12 y [25]. However, although sexually mature at 12 y of age, wild male walruses are believed to not be able to successfully reproduce until sometime after age 14 when physical maturity is reached [3, 25]. Clearly reproductive maturation precedes physical maturation, with successful breeding in both captivity and wild populations dependent on physical dominance of rival males and estrus females. Therefore, the earlier age at reproductive success for our males may be a result of less inter-male competition in captivity compared to their wild counter parts.

Using serum progesterone concentrations to indicate ovulation in females, we determined a mean age at first ovulation, also defined as reproductive maturity, as 8.8 y (range 5.8 to 12.8 y) with first conception at 9.6 y. These results were similar to results within a smaller group (4 females) of zoo-based females that found the earliest age at ovulation occurs at 6 y [19, 26]. For wild Pacific walruses, post-mortem ovarian corpora lutea counts indicated that reproductive maturity occurs from 5 to 8 y of [18, 19], while Atlantic walruses have been found to become pregnant as early as 5 y [18]. The age delay of almost a year between reproductive maturity and first conception in captive Pacific walruses may be a phenomenon created by *ex situ* environments whereby females do not always have access to mature males when physiologically able to breed. Our observation of the oldest age at calving for a female in our population was 28 y, despite females living well beyond 30 y of age, provides evidentiary support for the existence of reproductive senescence in this species. This observation is similar to what has been reported for wild walruses whereby reproductive rates for females was described as essentially zero by age 30 [13].

### Growth and development

The observed weight of captive born neonates which ranged from 49 to 76 kg is similar to data from wild calves which has been reported to be between 45 and 75 kg [3]. In addition, and like reports of sexual dimorphism in wild walruses [14, 27], males gain weight more rapidly than females with a linear growth rate for males (99 kg/year) at almost twice that of females (58 kg/year). These differential growth rates resulted in a permanent divergence in weight by age 5 y with mean adult weight for females (946 kg) occurring by 12 y and males (1381 kg) by 14 y. For wild Pacific walruses, females are described as reaching an estimated adult weight of 830 kg by age 12, and males are predicted to reach adult weight (1200 kg) after age 16 [3]. However, males are also described as having a secondary weight gain acceleration between 10 and 16 y prior to reaching adult weight [3]. Although we did not observe any secondary change in weight, we did observe a significant seasonal variation in weight associated with T concentrations. If wild males do not have an accelerated growth period as had been previously proposed, the large seasonal change in weight may be one reason why it was erroneously identified. For example, unequal sampling of wild males across the year could result in the appearance of a secondary growth spurt when in fact it was a result of the seasonal change in weight related to breeding (i.e., rut).

As with weight, and has been reported for wild walruses [3], length was similar at birth between sexes, with males becoming significantly longer than females by age 10 and asymptotic length occurring for females (276 cm) and males (323 cm) at 10 and 13 y, respectively. Similar to wild Pacific walruses [3], we found mean adult length for females greater than 9 y was 272 cm and 320 cm for males greater than 14. In wild walruses, although regional differences may exist between Atlantic Canadian or Norwegian populations, the overall evidence suggests that Atlantic walruses are slightly smaller in total length than Pacific walruses with adult length in females reported to be from 259 to 264 cm and from 304 to 317 cm in males [23, 27]. However, more recent work has shown adult (asymptotic) length for Atlantic females walruses from Northeastern Canada longer than those reported herein and for the wild female Pacific walruses at 276 cm, but male length, at 315 cm, is slightly smaller than our results [14].

### Seasonality and the reproductive cycle

Only one estrus occurred for each individual within this group of Pacific walruses during February to June with the majority in March. The observation of only a single estrus further substantiates evidence from other species that pinnipeds are generally annually monoestrous [28]. Although, Born et al. [18] reported that female Atlantic walruses can ovulate multiple times during estrus, we did not perform ovarian exams to determine if multiple ovulations regularly occurred within our subjects; however, with the exception of one female that repeatedly had twins, and because walruses rarely produce twins [29], it seems most likely that single ovulations during each estrus are the normal occurrence.

A single estrus per breeding season, as was observed herein, is similar to the Atlantic walruses whereby a single estrus occurs sometime from mid-January to late June and wild Pacific walruses where breeding has been observed sometime during late January through at least March [3, 18]. That the timing of estrus appears to be relatively similar within this species, regardless of large latitudinal changes, supports a hypothesis proposed for fur seals that if photoperiod is important for regulating seasonal reproduction, pinnipeds are sensitive to any seasonal change regardless of magnitude of that change [30].

With walruses, photoperiod may not tightly regulate estrus, but for other pinnipeds, it does appear to be crucial for tight regulation of their annual reproductive cycle. In order to maintain an annual reproductive cycle, seals (Family *Phocidae*) must have a short period of pupping and lactation and an almost immediate post-partum breeding and conception; thus, they may have evolved with a greater dependency on large changes in photoperiod to stimulate a more precise control of reproductive timing [28]. Evidence for this possible dependency on the magnitude of photoperiodic change to regulate reproductive events in phocids may be found by comparing the compact reproductive cycle of high latitude phocid species with the diffuse reproductive seasonality found within the only subtropical (~ latitude 20° N) phocid species, the Hawaiian and Mediterranean monk seals [31, 32]. Therefore, within the diverse physiology of reproduction found in pinnipeds, evidence presented herein and elsewhere for walruses support the concept proposed for fur seals [33, 34], that if photoperiod is important for regulating reproduction, it must integrate with both genetic and other seasonal environmental influences.

We observed significant annual changes in male and female body weight similar to what has been described for wild walruses and other pinniped species [3, 15, 35–37]. For male pinnipeds, seasonal increases in weight are hypothesized to be triggered by photoperiod and believed to be necessary preparation for the energetic costs encountered during the polygynous breeding season, but it is less clear what is the primary driver for weight gain in females [38–40]. Boyd (1984) postulates that endocrine changes, as experienced in false pregnancy, or at the time of implantation, may influence fat metabolism and seasonal weight changes in female pinnipeds. Our results also demonstrated that non-pregnant females experienced a significant seasonal weight change, but as expected pregnant females deviated significantly from non-pregnant females in weight gain by three months post-implantation (or October, assuming a March conception) that continued through parturition. For non-pregnant females, the increases may have been initiated by hormonal changes post-ovulation and false pregnancy, however, peaks in weight occurred months after the progesterone influence would have been removed. Further research will be required to understand the primary drivers for these metabolic changes.

For males, serum T fluctuated annually in tight synchrony with body weight, indicating a possible congruent metabolic and endocrine response to photoperiodic changes. Additionally, the anabolic effect of androgens may play a direct synergistic role in regulating the metabolic changes that result in an increased body weight [41, 42]. Although serum T was the primary androgen measured in this study, other endogenous androgens, especially 5α-dihydrotestosterone, are known to have an even greater anabolic effect [42–44] and future studies should be conducted to determine if they are present in significant amounts in the walrus.

In seasonally breeding polyestrous or monestrous mammals a pre-breeding rise in circulating T is commonly observed and is required to initiate spermatogenesis prior to the female receptive period [45]. The time required from initial T upregulation to the presence of mature epididymal spermatozoa represents the spermatic cycle or time required for upregulated spermatogenesis to result in mature sperm cells within the epididymis to be available for fertilization. We observed peak T in males in January, which was 45 to 60 d prior to peak fertility as determined by conceptions. Although conception is an indirect and imperfect measure of peak sperm production, the lag time we observed fits within the known range of spermatogenesis of 38 to 72 d across a wide group of mammalian species [46–48]. Testosterone concentrations dropped precipitously by April and were significantly reduced by July and August, corresponding with a significant decrease in body weight.

### Pregnancy and false pregnancy

We found a gestation length of 423 d which is similar to that which has been predicted for wild populations and we observed an approximately 3-year calving cycle for females which nursed calves [18, 19]. Interesting, a 2-year calving interval has also previously been proposed [3], which we only observed in females without live or suckling calves. Therefore, observation of a wild female with a calf two years after it was previously observed with a calf, would be a strong indicator of calf loss. A 423-day gestation (or ~ 14 month) is consistent with an offset between the breeding season or observed conceptions and birth, whereby peak conceptions occurred in March (ranging from February to June) and with peak births in May (ranging from April to June). A 14-month gestation length is less than the 15 to 16 month period that was originally predicted by Fay [3, 18, 49]. Differences between our and Fay’s estimates appear to be related to our approximate 10-month post-diapause development phases versus Fay’s (1982) prediction of an 11-to-12-month period.

In addition to gestation length, we detected significant monthly differences in serum progesterone (P) concentrations between pregnancy and false pregnancy. Based on this analysis, and similar to an estimated date of implantation for wild Atlantic walruses [18], we predict that the deviation in P4 during July (conceptions aligned in March), or approximately 120 d post ovulation, represents the point for pregnant females when implantation begins to occur.

Debate exists as to whether the length of diapause is controlled or adjusted by adherence to a photoperiodic induced implantation date, or if the ~ 120 day period we observed is a fixed physiologic requirement prior to reactivation [33]. If walruses did not have a periodic plasticity for the length of diapause prior to a relatively fixed blastocyst reactivation date one would expect a large range of implantation dates that mirrored or were consistently offset from conception dates. Thus, if true, blastocyst reactivation dates for walruses would occur from June until October. In addition, because gestation lengths in species without embryonic diapause is more defined [50], a fixed physiologic reactivation date would then translate into a birthing interval that mirrored the conception date window of 5 months. However, we observed a three month or 40% compression in the observed range for births compared to conceptions. This 40% compression in birth month period supports the concept proposed for fur seals [33], that in walruses the time of blastocyst reactivation is influenced by photoperiod and this effects the length of embryonic diapause such that animals conceiving early within the season would have an increased length in diapause while the period for those that conceive late would be compressed. The synchronization of blastocyst reactivation is believed to be important for ensuring that calving occurs during environmental conditions, e.g., the presence of sea ice, that are more advantageous for calf survival.

Although we could not accurately determine the total length of false pregnancy due to insufficient longitudinal samples from each individual, we were able to estimate a minimum length of 231 d (169 to 293 d), a duration that is well beyond the estimated point of implantation (~ 120 to 140 d post ovulation). Similar to our results, false pregnant harp seals are described as having peak progesterone in July which then gradually decreases over the following 4 months [21]. A four to five month period of elevated progesterone without conception has also been reported for harp and harbor seals [21, 22, 26]. The etiology of false pregnancy varies among species, whereby it is considered an obligate consequence of non-fertile ovulation in the dog, or pathologic in origin as is the case for most domestic species and in humans [51–55]. In dogs in response to the prolonged progesterone exposure and in humans, primarily psychologically induced, various changes can occur that mimic signs of pregnancy including weight gain, uterine hypertrophy, uterine milk secretions, lactation [52, 54–56], and physiologic and behavioral changes that inspired the term false pregnancy or pseudocyesis. False pregnancies as indicated by abnormally elevated progesterone, also known as persistent corpora luteal (CL), have been described in multiple species and is often believed to be the result of undetected early embryonic loss [57–60]. For walruses, the minimum length of the luteal phase or diestrus appears to be timed to coincide with of the length of embryonic diapause, with implantation believed to be either stimulated by a secondary ovulation as has been proposed for other pinnipeds and/or increased secretion of luteotropic factors from uterine, placental hypothalamic axis which then stimulates CL growth and which are required for CL maintenance during gestation [61–64]. Some debate exists for pinnipeds concerning whether or not the CL remains as the primary source of progesterone or that this role is eventually superseded by placental progestagen production [21]. Recent evidence using liquid chromatography-mass spectrometry indicated, that similar to dogs and Steller sea lions (*Eumetopias jubatus*), progesterone is the primary progestagen throughout pregnancy in walruses [65]. Because the CL is known to be active throughout walrus pregnancy, it stands to reason that the CL is the primary source of the circulating progesterone in the walrus, and the placenta provides little progestagen support during pregnancy [65], and, possibly, its role is primarily limited toward the production of a luteotropic hormone [66]

**Fig. 6:**
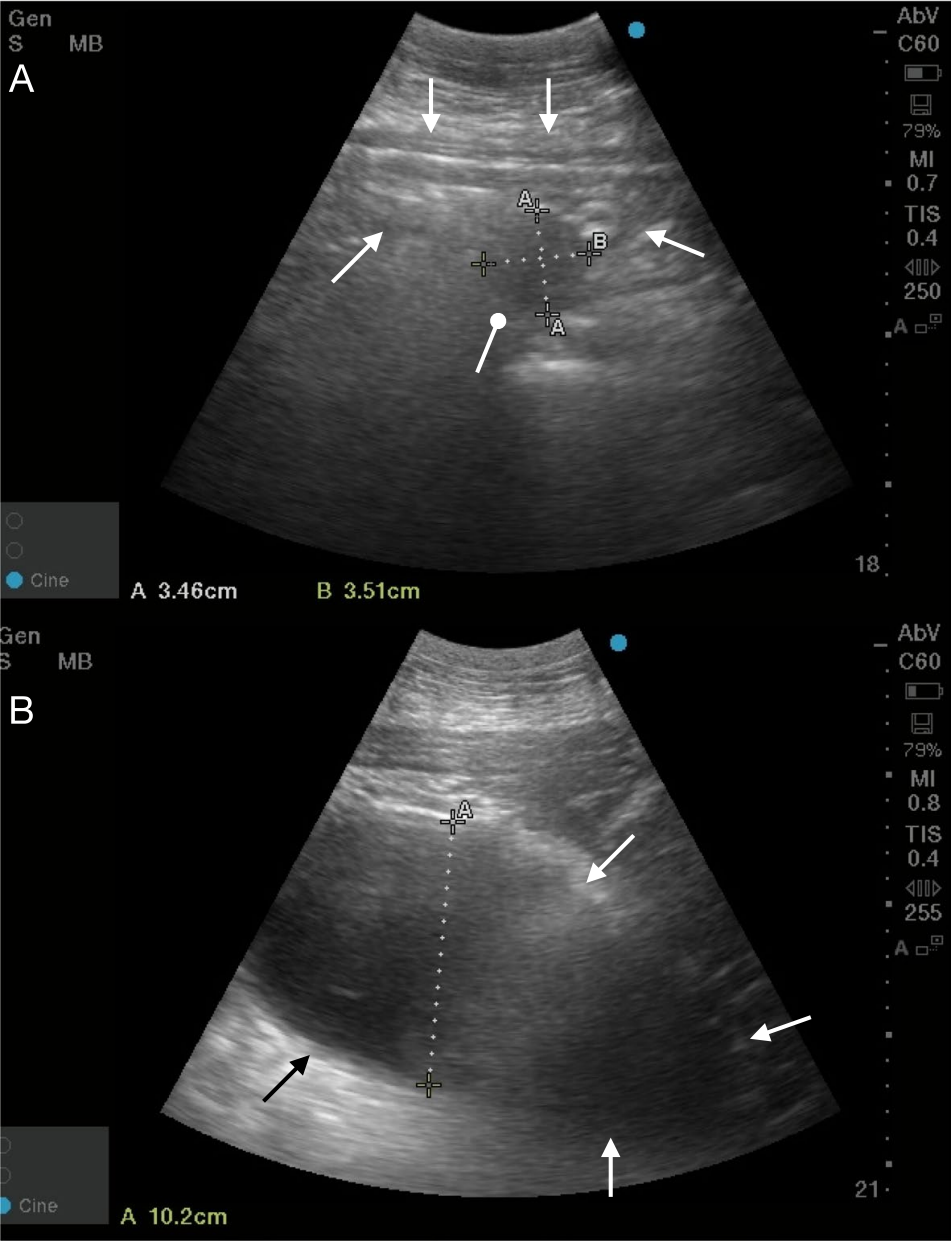
Ultrasound images during false pregnancy (FP) in a walrus. **A** Corpora luteal (round headed line) of false pregnancy shown within dotted crossed lines. The border of the ovary is highlighted by white arrows. **B** Fluid identified within the uterine horn — dilated to 10.2 cm of a FP walrus. The uterine horn is outlined by 3 white and 1 black arrows

Although progesterone is maintained post-ovulation until at least the estimated point of embryonic reactivation regardless of whether conception or copulation occurs, the wide variation in time before complete regression of the CL that occurs after this point in non-pregnant females indicates that other factors beside a lack of luteotropic support is affecting the length of the retained CL. As mentioned previously, for other species, one reason that these variable prolonged periods of elevated P4 may occur in, at least, females with access to breeding males, could be embryo or conceptus lost shortly after implantation and placental expansion has begun, but prior to a point whereby the pregnancy could be confirmed using transabdominal ultrasound or through observations of an aborted fetus. The loss after implantation could allow for the presumed initial surge of luteotropic activity, which would have stimulated CL growth possibly causing prolonged retention after the pregnancy loss. One sequalae of prolonged P4 secretion could be an increased likelihood of a physiologic and behavioral pseudo-pregnancy developing. In support of this theory, we have recently observed lactation and uterine swelling without evidence of conceptus via ultrasonography in a female walrus, that was observed breeding with a male, with P4 above baseline for 12 months after ovulation (unpublished observations). In another example, we presumptively diagnosed a female as pregnant based on elevated P4 beyond 140 d post ovulation and observable uterine fluid. However, as the “presumptive” pregnancy progressed, progesterone began to fall from the peak and at no time were fetal tissues observed (Fig. 6). This condition resembles false pregnancy in goats which is often linked to hydrometra, a common (~ 8%) pathologic condition in goats after non-conceptive ovulations or early embryonic loss [67, 68]. Ultimately, more investigations using ultrasonography in walruses that are potentially pregnant or have FP will help identify how often and under what circumstances this condition may develop.

A recent study reported pseudopregnancy in wild Alaskan walruses in May by identifying two females with CLs but no gross signs of pregnancy, and elevated concentrations of P4 that were significantly increased over a concentrations of progesterone within CLs from confirmed pregnant females [69]. However, with a reproductive tract collection date of early May, these females were most likely in the early pregnancy period whereby the developing embryos could easily be missed during gross examinations and when progesterone secretion is peaking. In support of this idea, Fay [3] states that all females with functioning CLs do not have grossly visible conceptus in the uterus during early pregnancy – a period that would encompass February into May [3]. Fay [3] provided supportive evidence by flushing uteri from two females that were harvested on May 14^th^ and May 30^th^ and microscopically identifying two blastocysts of 0.4 and 0.9 mm in size, respectively [3]. Blastocysts of this size would go unnoticed during a gross exam that did not include careful uterine flushing, filtering, and microscopic examination of collected fluid. The inability to grossly identify embryos in May has also been corroborated previously by multiple sources [18, 70, 71]. In addition, and again contrary to these recent observations [69], we observed that at no time were circulating concentrations of progesterone in false pregnant animals significantly increased over pregnant females during similar months post ovulation. These observed differences may have been a result of differing methodologies whereby Larsen Tempel et al., [69] measured progesterone in CL tissue while we measured circulating concentrations in the blood.

### Survival

We found neonatal calf survival to be of 53% and within the range estimated for wild calves (34 to 67%, [72, 73]). As expected, survival during the first 24 h was the most critical, with stillbirths accounting for 15.4% of all mortalities. If stillbirths were removed from the model, we found our neonatal calf survival surpassed the highest estimates for wild walruses at 86%. For older calves (from 90 d to 1 year), survival was 100% and surpassed all estimates for wild populations which range from 50 to 85% [72, 73]. Although we identified that most deaths occurred either at birth or within 24 h, the higher survival between neonates and older calves estimated for zoo-based walruses may indicate that the first 24 h post birth may be the most critical period for wild calves as well. If true, these stillbirths and early calf deaths would largely go unnoticed in wild populations, and thus account in large part for any disparity between wild and zoo-based animal calf survival rates. In addition, it highlights the importance of providing protective locations for walruses during the birthing season, and because climate change may affect or reduce available birthing locations (sea ice versus land), we need to better understand how, or if, these changes affect calf survival.

Our results provide the first direct measurement of survival using the Kaplan–Meier (KM) analysis within *ex-situ* walruses. Although we did not find a significant difference between male and female survival; male estimates were lower than females. For males, we found a median and mean life expectancy of 14.2 y and 17.4 y, respectively. Analyzing published population demographics from over 73,000 harvested walruses from multiple locations, an estimated median life expectancy between 10 and 14 y can be inferred, with only 28% of the male walruses living beyond age 14 [3]. Even though direct comparisons between our methodologies and these estimates are problematic [74], results from our ex situ population represent at least similar, if not improved, survival rates compared to wild males and may reflect an ideal, non-harvested or anthropogenically impacted, survivorship rate for a wild population. For females, the median and mean survival of 19.9 y and 20.7 y cannot be compared to historical data from harvested wild populations because published data from wild females were typically grouped based on reproductive potential and placed into one of three categories, immature (< ~ 6), reproductively active (6 to < 30) or post-reproductive ~ 30 or greater. Later work used an estimated 0.96 as annual survivorship rate (ASR) from 4 until age 39 to estimate potential population recovery rates. An ASR of 0.96 can be roughly translated to a median survivorship of 16.9 y [74]. Other recent efforts at determining ASR are also built around their applicability toward monitoring the effects of harvesting and environmental changes on population resilience and typically focus on ASR of adult animals. Therefore, they do not produce survivorship data across all age classes [72, 73]. Because survival does not remain steady over an animal’s lifetime, these results often overestimate median and mean survivorship rates. Primarily, our results provide a template for the potential life expectancy of the wild walrus populations that are not facing resource depletion and provide a measure from which other ex situ animals can be compared. In addition, we provide evidence that stranded or orphaned walruses live for significantly reduced periods of time when compared to wild caught and captive born. Although the reasons behind these differences probably vary with each case, in general, it does provide some evidence that rapid intervention of orphaned calves may be critical for their long-term health for either re-release or placement within a zoological setting. For maximum longevity, the oldest known living walrus is a female at almost 44 y, and multiple males and females have surpassed 39 y of age. Published estimates for maximum longevity of walruses have included 36 y for females and 35 for males [13, 14, 20, 23, 75]. Our data supports recent predictive estimates that walrus live for a maximum age of 40 to 44 y [73].

## Conclusions

Results from these analyses provide a comprehensive description of life history parameters of zoo and aquaria housed walruses. In some cases, the results are directly comparable to estimates reported for wild counterparts based on population statistics and provide validation though longitudinal observations of individuals as they progress through various life stages. In other instances, direct comparisons were not easily made due to the inability to identify and follow individuals from wild populations, for example, in survivorship model estimates. Therefore, our results on survivorship may be used as potential baselines from which wild population health can now be assessed. Finally, we provide evidence that the most critical period for successful recruitment of additional animals is within the first 24 h of life. This evidence can lend support for the critical need to protect birthing locations for this species.

## Methods

### Walruses

Data collected for the determination of growth, reproductive and seasonal parameters were collected from walruses (*n* = 30 walrus, Tables 1 & 2) located at three SeaWorld (SEA) facilities in the United States (6 male, 14 female) and one facility (Kamogawa SeaWorld) in Japan (KSW latitude 35.12° N), 5 male, 5 female). SEA facilities had a combined mean latitude of 30.25° N. Individually, these facilities included SeaWorld of Florida (latitude 28.54^o^N), SeaWorld of Texas (latitude 29.46^o^N) and SeaWorld of California (latitude 32.76^o^N). Because this research was a retrospective analysis (1980 to 2019) that relied on the availability of both stored serum samples for hormone analysis and historical data records, not all animals from each facility were used during each part of the analysis (Table 1). Blood samples were collected voluntarily from the hind flipper in a inter digital or metatarsal veins or from the inter vertebral sinus. Serum was obtained from blood samples and stored frozen (either -20 or -80° C) until hormone analysis.

### Life history data

We combined data from walruses from three SEA and one KSW facilities to determine age and weight at sexual maturation, defined as first ovulation in females and semen production in male, age at first conceptions and first and last births, gestation length, and seasonal distributions of mating and calving.

Ovulation was determined to have occurred based on elevation of serum progesterone. Blood samples were typically collected monthly during February to May for analysis of serum progesterone. In addition, breeding behavior between males and females were noted within behavioral records. If progesterone increased above baseline, blood collection was increased to as often as twice monthly. Initially, during the study period, pregnancy was confirmed by the continued weight gain of the female and the delivery of a calf. However, starting in the mid 1990s, transabdominal ultrasound examination was routinely used to confirm pregnancy via fetal detection [76]. The day of ovulation was defined as either the midpoint during which breeding activity was observed which resulted in an increase in progesterone concentration of greater than 1 ng/ml, or the midpoint between the last baseline sample and the first increased progesterone during the breeding season. If this period was greater than 60 d, no conception date could be determined.

We defined stillborn calves as term calves born dead or that died within 24 h of birth [77, 78]. Calves were considered born alive if they lived beyond 24 h. We also compared absolute date of calving in relationship to the spring equinox (assigned as Mar 20 of the corresponding year) in d between the SEA facilities and KSW using two-sided t-test with Welch’s approximation for unequal distributions.

To describe calf survival rates (CSR), we used calving dates from the Association of Zoos and Aquariums [79] inventory combined with the zoological inventories from SEA and KSW (duplicate entries removed) for all animals born at these facilities after January 1, 1980 until July 31, 2020. From these data, calf survival was estimated for five different sub-groups including: survival from 0–90 d and 0–365 d including and excluding stillborns and calf survival 90–365 d. (Supplementary File 3). The first subgroup included all term calves (both live and stillborn) and estimated calf survival for the 90-day period from birth to 90 d. Specifically, we first calculated daily calf survival (DCSR [80]) for this group using the equation below (Eq. 1) and then determined period survival by exponentiating daily survival with 90 d. The annual calf survival (ACS) was determined for the second subgroup by including all term calves from birth to 1 year of age. Similar, to the first subgroup, we first calculated DCSR for this group using Eq. 1 and then extrapolated daily survival to the ACS by exponentiating daily survival with 365. The third subgroup included only live calves from birth to 90 d and calculated the 90-day survival as described for subgroup 1. The fourth subgroup included live calves from birth through 1 year of age or 365 period d and was calculated as described for subgroup 2 above. Finally a fifth subgroup, termed older calf survival, defined as calves surviving from 90 to 365 d of age, was added for direct comparison to previous publications for wild walruses [72, 73]. This group was similarly determined by first calculating the DCSR using Eq. 1 and then extrapolating the period survival by exponentiating DCSR with 276 [80].

1 $$DCSR=1- \sum_{i=1}^{K}(Yi)/\sum_{i=1}^{K}(Xi)$$

where the summation of $Yi$ is the total number of calves that died during the observation period, the summation of $Xi$ is the total number of animal days observed, including any day in which death may have occurred, and K is the total number of animals in the sample.

### Growth rate

Weight (WT, kg) and total body length (TL, cm) were collected from 12 wild born (5 males and 7 females) and 14 captive born walruses (8 males and 8 females) during May 1978 until August 2019 (Table 1 & 2). TL was defined as straight line length (cm) from nose to tail, collected while the animal was in ventral recumbency, and WT (Kg) was measured using an electronic scale. To compare growth rates and final weight or length between male and female walruses, we used a non-linear mixed model analysis with individual animal (ID) as the random variable, weight or length as the dependent variable, age (d) as the independent variable and sex as a covariate.

We then fit a 3-parameter Gompertz growth model, either for combined sexes or each sex independently based on the results of the initial analysis, to describe growth (WT and TL) at age. The Gompertz model was chosen based on its previous use in marine and other mammalian species [81–84]. The model (1) is expressed as:

2 $$\normalsize WT\ or\ TL = \mathrm{ Aexp}(-\mathrm{exp}(-{\mathrm{k}}_{\mathrm{G}}(\mathrm{age}-{\mathrm{T}}_{\mathrm{i}})))$$

where WT or TL is weight or total length of the animal by age, A is the upper asymptotic weight or length (adult weight or length), k_G_ is the growth-rate coefficient and T_i_ is the timepoint at curve inflection[83]. Maximum relative growth rate (R_G_: at inflection point and relative to maximum value) was determined by dividing k_G_ by the natural logarithm (R_G_ = k_G/_*e*) and absolute growth rate (ABS_G_) was calculated by multiplying the upper asymptotic (A) by R_G_ [ABS_G_ = A(R_G_)] [83]. For the repeated measures of weight versus age from each animal, weight and age were assumed to be autocorrelated. The model was run using a bootstrapping technique (reps 1000) to estimate the standard errors (SE) and 95% confidence intervals (CI) [81, 82]. To adjust for autocorrelation, the variance–covariance matrix of the estimates (VCE) was calculated using the “cluster” option around animal ID. For length versus age, the frequency of measurements (medium 1.5 per year/per animal) reduced the problem of autocorrelation, nonetheless, the data were treated as if clustered around each animal, and due to the small sample size, Jackknife estimation was used to estimate the variance [85]. If it was determined that sex differences in weight or length existed, we then determined at what age these differences deviated permanently by first observing at what age non-overlapping 95% CI were detected (for example, age 4). We then ran a marginal mean comparison of weight or length (dependent variables) during each year of life across this estimated age of deviation (for this example, 0 to 6 y) using a mixed effect restricted maximum likelihood (REML) regression model [86], with ID as the random variable and age (year), sex and their interaction as factorial fixed effects variables.

### Seasonal weight changes

To determine if seasonal monthly weight changes occur in non-pregnant adult females and to compare their weight against changes occurring during pregnancy the following parameters were defined. First, weight data (kg) were used only from mature females defined as ≥ 8 y. This was based on evidence that the majority of wild female walrus have ovulated by age 8 are thus considered mature [3, 13]. Next, because gestation length is greater than one year (~ 14.5 months [15]), we only compared weight changes over the last 12 months of gestation – under the assumption that the weight during the first 2.5 months of gestation would be indistinguishable between pregnant and non-pregnant walrus. We used June (i.e., the month when all births occurred) as the point for alignment, and then compared weight between the two groups from July of the preceding year until June for the year of birth. Once a female gave birth, they dropped out of the comparisons for a minimum of 3 months post-partum to eliminate the weight from the post-partum uterus and the dramatic weight loss post-partum that occurs from the combination of birth, uterine involution, and energetic demands of early lactation. For example, if a female gave birth in May, her June–August weight was not included as a nonpregnant female data point. We then compared the mean monthly weight (kg) of pregnant females to nonpregnant females from June until May. A 2 or 3 level REML regression model quantifying the relationship between the dependent variable (animal weight) and fixed effect variables (level 1 of the REML, month post ovulation, animal age, and pregnancy status [coded 0 or 1]) and the random effects variables, animal ID (level 2 of the REML) and the pregnancy number (level 3 or the REML, each pregnancy was assigned a unique identifying number). Pregnancy number was used, if necessary, to control for any significant variance associated with variability in the number of pregnancies per animal. First, we determined if a 2 or 3 level REML accounted for significant variance of the dependent variable by comparing estimates of the 2nd and 3rd level models using the likelihood-ratio (LR) test at *p* < 0.05. Once the optimum random variables were determined (e.g., two or three level multilevel), all fixed effect variables were added to the model.

### Hormone analysis

Serum progesterone and testosterone were analyzed using an immunoenzymometric assay (TOSOH Bioscience NV, Tessendero, Belgium, progesterone: ST AIA PROG II; testosterone: ST AIA Pack Testosterone). Assay validations for this species included sample parallelism and accuracy. Parallel displacement of walrus sera compared to the standard curve for progesterone was demonstrated (*r* = 0.989), and the recovery of known concentrations of standard added to a pool of sera was 86.8 ± 14.1% (linear regression, y = 1.05x = 0.57, r2 = 0.995), thereby demonstrating negligible matrix interference. For progesterone, cross-reactivity of other hormones, according to the manufacturer’s directional insert, was: 17α-hydroxyprogesterone, 0.26%; 11-deoxycorticosterone, 0.81%; pregnelone, 0.49%; corticosterone, 0.001%; 5β-pregnan-3,20-dione, 0.06%; testosterone, 0.01%; cortisol, 0.002%; 5α-pregnan-3,20-dione, 3.78%; DHEA-S, 0.008%. For testosterone, parallel displacement of walrus sera compared to the standard curve was demonstrated (*r* = 0.998), and the recovery of known concentrations of standard added to a pool of sera was 99.5 ± 5.6% (linear regression, y = 0.99x – 1.52, r2 = 0.999), For the testosterone assay, reported cross-reactivity, according to the manufacturer’s directional insert, was: androstenedione, 1.71%,; androsterone, 0.03%; aldosterone, not detected (ND); corticosterone, 0.01%; cortisol, ND; cortisone, ND; danazol, ND; 11-deoxycortisol, ND; dexamethasone, ND; DHEA, 0.1%; DHEA-S, ND; 5α-dihydrotestosterone, 0.81%; estradiol, ND; estrone, ND; ethisterone, 0.02%; fluoxymesterone, ND; 19-hydroxyandrostenedine, 0.04%; methyltestosterone, 0.15%; norethindrone, ND; prednisone, ND; progesterone, 0.02%; norethynodrel, 0.03%; spironolactone, 0.01%; triamcinolone, ND. The limit of detection, according to the manufacturer was 0.05 ng/ml for progesterone and 7 ng/dl for testosterone. Intra-assay variation for both hormones was < 10%. Inter-assay variation for three quality controls (high, medium, and low) was 14.4, 11.1 and 10.8%, respectively (*n* = 57) for progesterone and 9.9, 12.6 and 12.5% (*n* = 38) for testosterone. Routine internal quality control checks were performed daily, and sample reanalysis was performed whenever an outcome outside of the expected range for the respective stage of the reproductive cycle was observed.

The actual total length of time for false pregnancies (FP) was difficult to ascertain due to the infrequency of sampling from animals after the breeding season, but to obtain an estimated length, we used the halfway point between last sample that had an above baseline progesterone (P) concentration (> 1 ng/ml) and the first sample that was continuously below baseline at the end of the FP period. Only samples that straddled these two P4 points and were separated by ≤ 60 d were used for this calculation. For example, the end of FP was determined to be the halfway point between two consecutive samples that were ≤ 60 d apart in duration, in which P4 concentrations decreased from above baseline to below baseline. Hormone concentrations in pregnant and FP females were then binned within months post ovulation (e.g., d 0 to 29 represented month 0 post ovulation [MPO], d 30 to 59 as MPO 2, etc.), and for graphical display and discussion purposes month zero was assigned as March, the month the majority of conceptions occurred.

For mean monthly hormone concentration comparisons within a pregnancy and between pregnancies, FP and non-pregnant, a 2 or 3 level REML was used with ID (level 2) and/or event ID (EID, Level 3, unique code assigned to each pregnancy or FP) as the random variables as determined by estimated comparisons of models with and without the EID as the level 3 using the LR test and *p* < 0.05. In addition to the dependent variable (progesterone), each model contained the appropriate fixed variables, month post conception (MPC), MPO, and/or status (non-pregnant, pregnant, FP: coded 0, 1, 2) and interactions (e.g., MPO*status if status > 0 for MPO comparisons between pregnant and FP). The continuous variable age was included in all models to account for any age associated changes in hormone production. The first analysis was conducted to compare for inter-monthly pregnancy progesterone concentrations aligned at month post-conception (MPC) across the entire pregnancy and a second one to compare monthly differences post-ovulation (MPO) between pregnancy and FP.

All final mixed effects models were checked for normality using quantile plots of the standard residuals. If quantile–quantile plots of standardized residuals exhibited non-normal distribution the data were transformed as predicted by the Shapiro–Wilk test (Ladder command, STATA) until residuals were normalized. Multiple comparisons of marginal means were performed post-hoc using Sidak corrections at *p* < 0.05. For text, tables and graphs any transformed data were first back transformed, and then all data were presented as marginal means with 95% confidence intervals (CI) unless noted otherwise.

### Life expectancy

Kaplan–Meier modeling (KM) of daily survival rate for captive walruses was performed using animal life span data from the AZA inventory combined with data from SEA and KSW (duplicate entries removed) for all animals alive or added to facilities after January 1, 1980 until July 31, 2020 (Supplementary File 3). The start date for the analysis was chosen because for many marine mammal species, the commencement of the modern era for animal husbandry, record keeping, training and facility design is considered to be in the early 1980s [74, 87, 88]. Furthermore, we limited our KM analysis to walruses > 6 months of age because the mortality rate of walrus calves is considered to be higher in the first few months in wild populations, and is somewhat difficult to predict or observe [72, 73]. Due to this variability, survival rates are typically calculated for wild populations starting at age 1 y. However, due to our ability to accurately monitor animals of any age, we started our analysis with animals at 6 months in age [13, 74, 88]. Finally, we determined survival for the overall sample, and then, the sample was stratified by sex and then into subgroups as follows: captive born; wild born collected (wild born – animals collected as neonates within the calving season); or rescued (beached – brought into captivity greater outside of the calving season). Walrus calves were classified as beached or wild caught based on records at collection. The KM analyses was performed using Stata statistical software (StataCorp, ver 16.1). Stata can handle delayed entry (left truncated, or animals that enter the sample at different ages) of individuals into the sample. We used the log-rank test [89] for subpopulation comparisons of equality and to calculate 95% confidence intervals in both mean and median life expectancies.

## Supplementary Information

**Additional file 1.** 

**Additional file 2.** 

**Additional file 3**. 

## Data Availability

All data generated or analyzed during this study are included in this published article and its supplementary information files.

## Abbreviations

SEM: Standard error of the mean
CI: Confidence interval
MSE: Mean squared error
P4: Progesterone
MPO: Month post ovulation
FP: False pregnancy
MPC: Month post conception
PrI: Pre-implantation
PI: Post-implantation
T: Testosterone
KM: Kaplan–Meier
CL: Corpora luteal
SEA: SeaWorld Parks and Entertainment
KSW: Kamogawa Sea World
CSR: Calf survival rates
DCSR: Daily calf survival rate
ACS: Annual calf survival
WT: Weight in kilograms
TL: Total body length in centimeters
ABS_G_: Absolute growth rate
VCE: Variance–covariance matrix of the estimates
REML: Restricted maximum likelihood
LR: Likelihood-ratio
